# Demystifying the Chemical Ordering of Multimetallic Nanoparticles

**DOI:** 10.1021/acs.accounts.2c00646

**Published:** 2023-01-21

**Authors:** Dennis Johan Loevlie, Brenno Ferreira, Giannis Mpourmpakis

**Affiliations:** Department of Chemical and Petroleum Engineering, University of Pittsburgh, 3700 O’Hara Street, Pittsburgh, Pennsylvania15261, United States

## Abstract

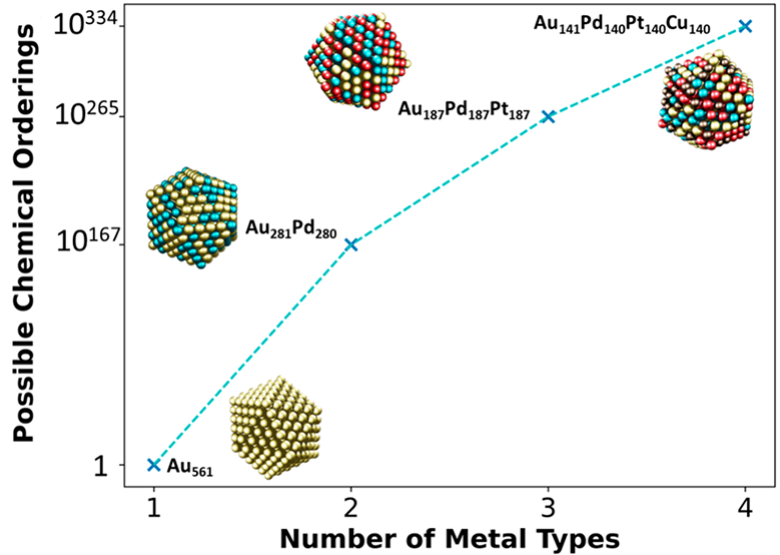

Multimetallic nanoparticles (NPs) have highly tunable properties due to the synergy between the different metals and the wide variety of NP structural parameters such as size, shape, composition, and chemical ordering. The major problem with studying multimetallic NPs is that as the number of different metals increases, the number of possible chemical orderings (placements of different metals) for a NP of fixed size explodes. Thus, it becomes infeasible to explore NP energetic differences with highly accurate computational methods, such as density functional theory (DFT), which has a high computational cost and is typically applied to up to a couple of hundred metal atoms. Here, we demonstrate a methodology advancing NP simulations by effectively exploring the vast materials space of multimetallic NPs and accurately identifying the ones with the most thermodynamically preferred chemical orderings. With accuracies reaching that of DFT, our methodology is applicable to practically any NP size, shape, and metal composition. We achieve this by significantly advancing the bond-centric (BC) model, a physics-based model that has been previously shown to rapidly predict bimetallic NP cohesive energies (CEs). Specifically, the BC model is trained in a way to understand how the bimetallic bond strength changes under different coordination environments present on a NP and how the metal composition of every site affects the detailed coordination environment using fractional coordination numbers. This newly modified BC model leads to an improvement from 0.331 (original model) to 0.089 eV/atom in CE predictions when compared to DFT values on a robust data set of 90 different NPs consisting of PtPd, AuPt, and AuPd NPs with varying compositions and chemical orderings. By incorporating the modified BC model into an in-house-developed genetic algorithm (GA) we can effectively and accurately predict the most stable chemical orderings of large, realistic bimetallic NPs consisting of thousands of metal atoms. This is demonstrated on AuPd bimetallic NPs, a challenging system due to the similarity in the cohesion of the two metals. By training our BC model using a unique DFT calculation on a bimetallic NP (one calculation for two metals combining together), we expand to explore the chemical ordering of multimetallic NPs. We first demonstrate the application of our methodology on a AuPdPt NP and validate our stability predictions with literature data. Then, we effectively explore the vast materials space of multimetallic NPs consisting of combinations of Au, Pt, and Pd as a function of metal composition. Our thermodynamic stability trends are presented in a ternary diagram revealing detailed, and yet, unexpected chemical ordering trends. Our computational framework can aid both experimental and computational researchers toward effectively screening multimetallic NP stability. Moreover, we provide an outlook of how this framework can be applied to catalyst discovery, high-entropy alloys, and single-atom alloys.

## Key References

- Yan, Z.; Taylor, M. G.; Mascareno, A.; Mpourmpakis, G. Size-, Shape-, and Composition-Dependent Model for Metal Nanoparticle Stability Prediction. *Nano Lett.***2018**, *18*, 2696–2704.^1^*The bond-centric model was introduced, and through comparison with DFT calculations, it was shown to capture the mixing (excess energy) of nanoalloys and the cohesive energy trends for a range of bimetallic nanoparticles*.
- Dean, J.; Cowan, M. J.; Estes, J.; Ramadan, M.; Mpourmpakis, G. Rapid Prediction of Bimetallic Mixing Behavior at the Nanoscale. *ACS Nano***2020**, *14*, 8171–8180.^2^*A genetic algorithm was developed, parametrized by the bond-centric model, to rapidly predict the most stable chemical ordering of any given bimetallic nanoparticle.*
- Dean, J.; Taylor, M. G.; Mpourmpakis, G. Unfolding adsorption on metal nanoparticles: Connecting stability with catalysis. *Sci. Adv.***2019**, *5*, eaax5101.^3^*A universal adsorption model on metal nanoparticles was developed based on surface site stability described through the bond-centric model. The adsorption model can capture the binding strength of small molecules on any site of bimetallic nanoparticles of any morphology and metal composition.*
- Cheula, R.; Maestri, M.; Mpourmpakis, G. Modeling Morphology and Catalytic Activity of Nanoparticle Ensembles Under Reaction Conditions. *ACS Catal.***2020**, *10*, 6149–6158.^4^*A methodology was introduced to describe the catalytic behavior of nanoparticle ensembles at elevated experimental temperatures through the use of nanoparticle stability models and Boltzmann statistics.*

## Introduction

Metal nanoparticles (NPs) have tremendous applications in various fields, including, among others, biomedical diagnosis and treatment,^5^ optical sensing and imaging,^6^ and catalysis.^7−9^ Focusing on catalytic applications, the metal NP composition and morphology, such as size and shape, play crucial roles in the catalytic activity, selectivity, and stability of NPs.^4,10−12^ In addition to the composition and morphology, it has been shown that chemical ordering (i.e., how different metal atoms are distributed in the NP lattice) has a large effect on the stability and electrocatalytic activity of bimetallic NPs in reactions such as oxygen reduction (ORR) and small-molecule (hydrogen, formic acid, or ethanol) oxidation.^13,14^

Recently, PdAu bimetallic NPs have emerged as promising catalysts for energy and environmental applications, such as the reduction of CO_2_ to CO (reducing the greenhouse effect) and the production of H_2_O_2_ (important reagent for the chemical industry). For instance, CO_2_ can be reduced in the presence of CH_4_ (from natural gas) to CO and H_2_ on PdAu NPs using visible light.^15^ In addition, PdAu alloy nanocrystals, with atomically dispersed Pd sites on the surface of the nanocrystal, show superior CO_2_ electrochemical reduction performance, with a 94% CO faradaic efficiency at −0.5 V (vs reversible hydrogen electrode) and approaching 100% from −0.6 to −0.9 V.^16^ The CO_2_ electrochemical reduction has also been investigated on an ultrasmall, Pd-doped Au_25_ nanocluster (protected with ligands), which converted CO_2_ to CO with ∼100% faradaic efficiency ranging from −0.6 (onset) to −1.2 V (vs reversible hydrogen electrode), whereas the pure Au_25_ nanocatalyst declined at −0.9 V.^17^ In all of these studies, the chemical composition of the NPs appears to be a critical factor for the CO_2_ reduction behavior. Ricciardulli et al. showed, through in situ X-ray absorption spectroscopy and quantum chemical simulations, that PdAu NPs can be promising catalysts for direct H_2_O_2_ production.^18^ The selectivity was shown to be highly dependent on both the composition, where higher Au to Pd compositions showed better selectivity (nearing 100% for Pd_1_Au_222_), and the chemical ordering, where PdAu NPs exhibited higher selectivity when Pd monomers were dispersed on the surface. Bimetallic NPs that contain Pt and either Au or Pd have been shown to be valuable catalysts for a plethora of reactions. Depending on the composition, PtPd NPs have been shown to have up to a 2.4 times better relative mass activity for the ORR when compared to monometallic Pt NPs.^19^ Shao et al. evaluated bimetallic PtAu NPs with different Pt to Au compositions and found that Pt-rich PtAu NPs exhibited enhanced catalytic activity for selective hydrogenation reactions of substituted nitroaromatics.^20^ It becomes apparent that the chemical ordering at different metal composition is crucial for the catalytic performance of bimetallic NPs. This is a general phenomenon and not limited to bimetallic combinations. For example, moving to trimetallic NPs, the PdAu/Pt NPs supported on CNTs have shown incredible catalytic performance and stability for the methanol oxidation reaction (MOR) with an electrocatalytic peak current of 4.4 A mg_Pt_^–1^.^21^ The total metal composition in addition to the chemical ordering of the metals on the NP dictate the surface composition, which is critical for catalytic applications since catalytic reactions occur on the surface of NPs.

Due to the importance of NP chemical ordering on the catalytic activity and stability of multimetallic NPs, it is crucial to develop computational methods that accurately determine optimal chemical orderings (i.e., thermodynamically most stable) at different NP compositions, shapes, and sizes. Monte Carlo (MC) simulations have been widely applied to determine the surface segregation and chemical ordering of metal NPs.^22,23^ Rahm and Erhart applied hybrid molecular dynamics–MC simulations to understand the equilibrium chemical ordering of PdAu and AgCu NPs.^24^ Li et al. used an adaptive kinetic MC method to study the surface segregation dynamics of PdAu NPs during experimentally relevant time scales due to the importance of the NP surface composition and local ordering in the catalytic activity.^25^ Alternatively, Kozlov et al. proposed a topological energy expression method which is parametrized by density functional theory (DFT) and allows for a global optimization of the chemical ordering in large bimetallic NPs.^26,27^ One issue with most of the empirical and semiempirical methods is that they require parameter fitting from large DFT data to accurately model nanoalloy energetics.^1,28^ Yan et al. developed a bond-centric (BC) model, which is an extension of a simple square-root bond-cutting (SRB) model,^29^ that rapidly and accurately predicts bimetallic NP cohesive energy (CE) values.^1^ The power of the BC model lies on the fact that it utilizes bulk metal CE values (which can be found tabulated), the coordination environment of the NP, and gamma values (i.e., parameters) that scale the CE of the monometallics to capture the bimetallic bond strength. As a result, it is very rapid to assess the CE of a NP of any metal composition, size, and shape. This BC model was further used to parametrize a recently developed mixed integer genetic algorithm (GA) that has been shown to accurately predict the most stable chemical ordering of bimetallic NPs with varying compositions, sizes, and shapes.^2^ The GA parametrized by the BC model has been shown to outperform (i.e., find more thermodynamically stable chemical orderings when evaluated by DFT) previous methods^30^ using mixed integer optimization parametrized with effective medium theory^31^ on AgAu NPs.^2^ Thus, the combination of accurate NP stability models, typically derived from DFT, with machine learning can very efficiently screen the tremendously large materials space of bimetallic NPs. The materials space increases with the size of the NP (i.e., total number of metal atoms) and the number of different metals comprising the NPs (see conspectus figure for a 561-atom NP). As a result, the complexity increases dramatically going from monometallic to bimetallic to multimetallic NPs. High-entropy alloys (HEAs) are multimetallic NPs that contain more than four elements uniformly mixed into a solid–solution structure and open an exciting, vast materials space for catalytic applications.^32^ These multimetallic NPs have been applied to a plethora of catalytic applications including ammonia decomposition where they show high stability and an increased reaction rate, almost 4-fold, in comparison to ruthenium (Ru) catalyst (at 500 °C).^33^ Toward understanding the stability of multimetallic systems, Clausen et al. developed a computational methodology for screening different alloy compositions to find optimal catalysts through the use of first-principles-based graph neural networks and Bayesian optimization.^34^ This method was applied to the ORR with an alloy composition space over the HEA Ag–Ir–Pd–Pt–Ru.

In this Account, a computational roadmap is provided that reveals the stability of multimetallic NPs of any size, shape, and metal composition. Although we demonstrate results on combinations of Pd, Au, and Pt metals for bimetallic and trimetallic NPs, our methodology is general and extendable to any metal composition. We first introduce our efforts to advance the previously developed BC model^1^ to capture information from the coordination environment of the NP on the bimetallic bond strength. We then propose a modified coordination number to capture strain effects on the CE of multimetallic NPs. Finally, we demonstrate the generalization of the BC model to multimetallic NPs and present the optimal chemical orderings of AuPdPt NPs over a large range of compositions.

## Optimal Chemical Ordering of Bimetallic Nanoparticles

We investigate the optimal chemical ordering of bimetallic NPs using a previously developed GA^2^ that minimizes the NP CE, as given by the BC model in eq 1, by rearranging the chemical ordering of metal atoms. To calculate the gamma values in eq 1, one needs to calculate the bond dissociation energies (BDE) of monometallic and bimetallic dimers^1^ (in this example study Au and Pd). The bimetallic gamma values (i.e., *γ*_A__–B_ and *γ*_B__–A_) are calculated according to eqs 2 and 3. The gamma values are weighting factors for the bimetallic bond strength between metals A and B. If the bond is between two atoms of the same metal type, the gamma values (i.e., *γ*_A__–A_ and *γ*_B__–B_) are both equal to 1. This method of calculating the gamma values will be referred to as the dimer method.
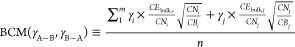
1
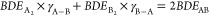
2
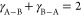
3In eq 1, *m* is the total number of bonds of the NP, *n* is the total number of atoms of the NP, *i* represents atom *i* (bonded to atom *j*), *CN*_*i*_ is the coordination number (CN) of atom *i*, *CB*_*i*_ is the bulk CN of atom *i* (i.e., 12 for fcc metals), and *CE*_bulk__,*i*_ is the bulk CE of atom *i*. BCM stands for the BC model and is dependent on the values of *γ*_A__–B_ and *γ*_B__–A_. In eq 2, the gamma (γ) values capture the proportional relationship between monometallic and heterometallic BDE’s.

**Figure 1:**
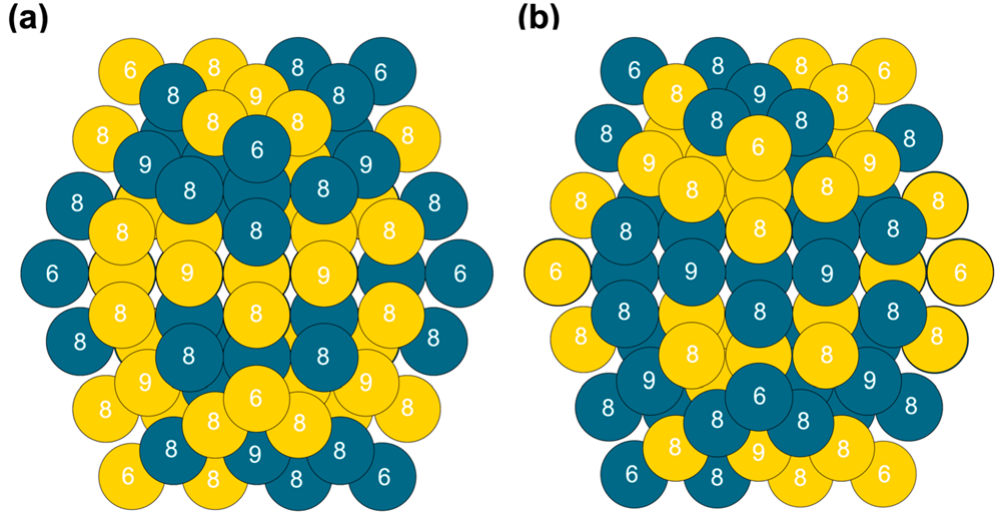
Visualization of the NPs used to calculate the NP method gamma values (with surface atoms labeled according to their CN to demonstrate the distribution of atoms in different coordination environments): NP composition of (a) Au_74_Pd_73_ and (b) Au_73_Pd_74._.

It has been shown that a square root bond-cutting model (SRB) can accurately predict the surface energetics for most (monometallic) transition metals.^35^ The BC model parametrized by gamma values calculated with the dimer method demonstrated increased accuracy compared to the SRB for bimetallic NPs over a large range of compositions and sizes.^1^ Herein, we propose a novel method for calculating the *γ*_A__–B_ and *γ*_B__–A_ values, referred to as the NP method, to capture the variation of coordination environments on a NP and its impact on the bimetallic bond strength that is not captured in the original dimer method from Yan et al.^1^ The difference between the proposed NP method and the dimer method is that eqs 2 and 3, which are used to calculate the gamma values with the dimer method, are replaced with eqs 4–6. Equations 4–6 are shown below and are analogous to the ones in the original derivation (eqs 2 and 3, with eqs 3 and 6 being identical), albeit the energy values are calculated differently. When calculating *CE*_A__,B,*n*_ the NP is 50/50 metal A and B (or very close to this composition if it consists of an odd number of atoms), and the two different metals, A and B, occupy a variety of coordination environments in an equal occupation maximizing the A–B heterometallic bonds. In the special case that the NP consists of an odd number of atoms, two NPs were taken into consideration as a reference with flipped compositions on metals (to maintain an equal distribution of metals on different coordination environments, see eq 5, *n*-odd case). An example of a Au_74_Pd_73_ and Au_73_Pd_74_ NP used in the DFT calculations to calculate the gamma values of NPs containing Au and Pd is shown in Figure 1a and 1b, respectively.

***n*-even**
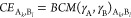
4***n*-odd**
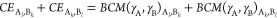
5
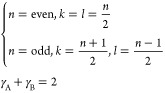
6

**Figure 2:**
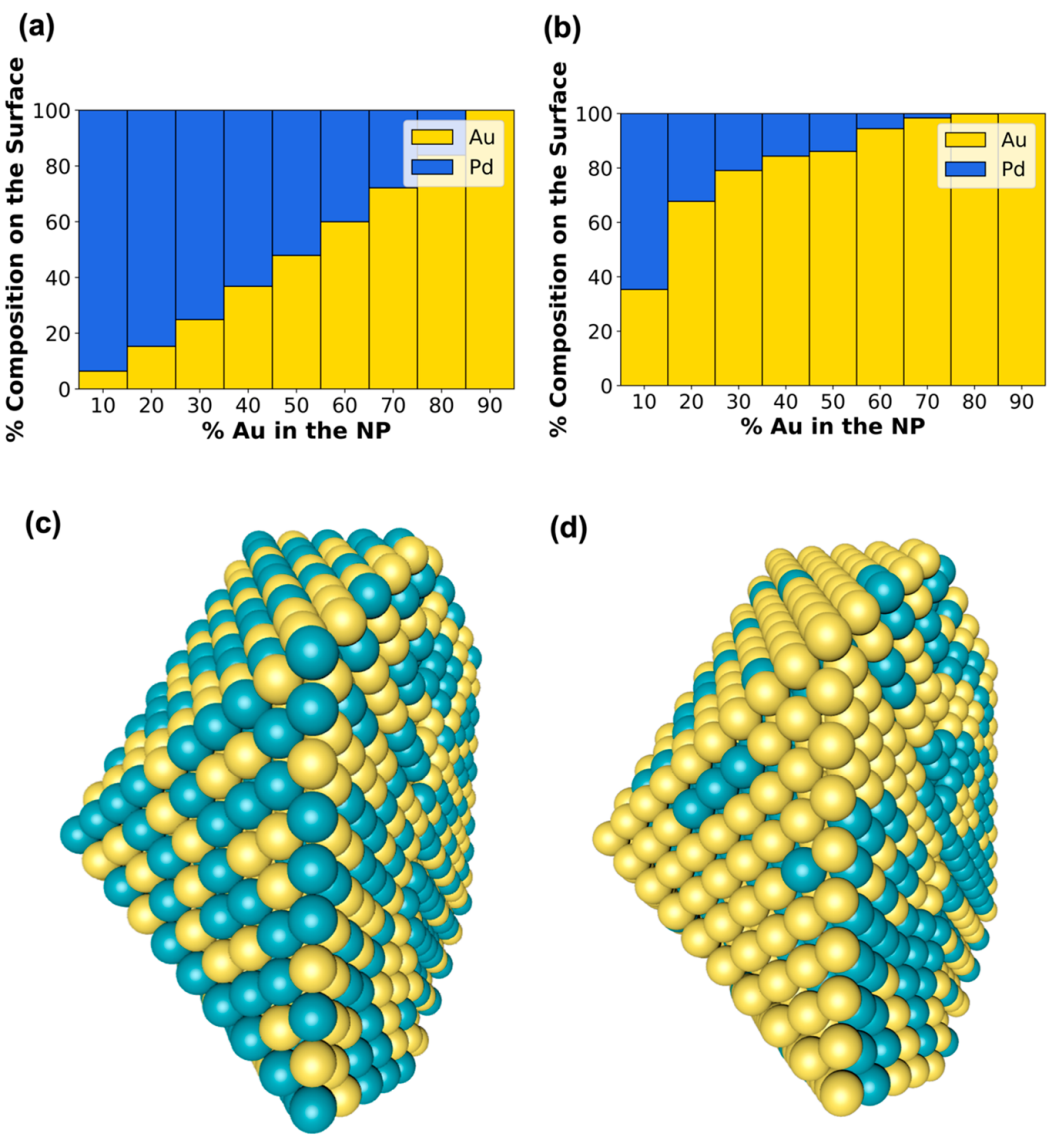
Surface composition of a 2869-atom icosahedron PdAu bimetallic NP at different compositions of Au using (a) the dimer method and (b) the NP method to calculate the gamma values. Center cut (along *x* axis) of 50/50 composition NP optimized using the (c) dimer method and (d) the NP method.

**Figure 3:**
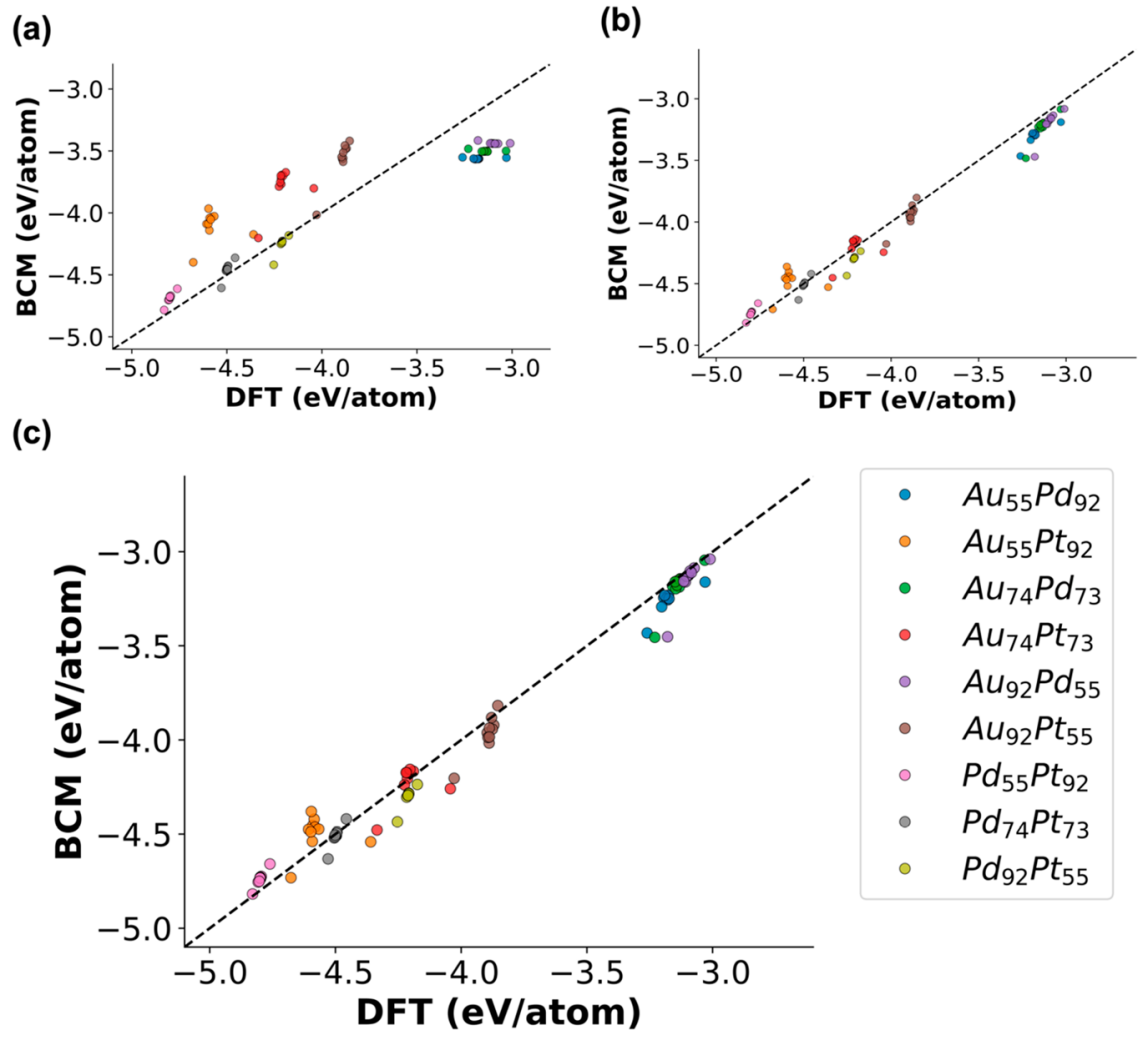
Parity plot between the BC model and the DFT CEs for different NP combinations of Au, Pt, and Pd at different compositions and chemical orderings: (a) Using the dimer gamma values and the original CN definition (RMSE 0.331 eV/atom), (b) using the NP gamma values and the original CN definition (RMSE 0.100 eV/atom), and (c) using the NP gamma values and *CN*_frac_ (RMSE 0.089 eV/atom).

The DFT calculations, used to geometrically optimize and find the CE of the NPs (i.e., binding energy per atom) from Figures 1 and 3, were performed with CP2K^36^ using the PBE functional.^37^ The D3 correction^38^ was used due to the bulk CE values for Au being much closer to the experimentally determined values (0.16 eV difference) than when using the PBE functional to determine the bulk CE values (0.8 eV difference).^39^ The GA was then used to optimize the chemical ordering of nine different NPs consisting of 2869 metal atoms each with different compositions of Pd and Au (this NP size is beyond the allowable sizes calculated with DFT, which is typically a couple of hundred metal atoms). This was done using gamma values calculated from the original dimer method, as shown in Figure 2a, and the developed NP method, as shown in Figure 2b. A center cut of the 50/50 composition NPs with optimized chemical orderings using the GA parametrized with the dimer gamma values and NP gamma values is shown in Figure 2c and 2d, respectively. The NP method tends to predict the surface segregation of Au in all cases, but the dimer method seems to flip this trend, predicting that the NP surface will be rich in Pd. Previous studies have found that Au prefers to segregate to the surface in PdAu NPs.^40,41^ Specifically, using MC simulations, Zhu et al. showed that Au tends to segregate to the surface due to the differences in surface energy and the size of the two metals.^41^ Similarly, Pernilla et al. showed that Au segregates to the surface (modeled as a ⟨111⟩ facet surface slab) through the use of alloy cluster expansions trained using DFT, MC simulations, and thermodynamic analysis.^40^ Therefore, the use of the NP method for calculating the bimetallic gamma values in the BC model results in physically meaningful chemical ordering predictions in agreement with literature studies. Although the dimer method is based on rapidly calculating the BDE of metal dimers and has shown robustness in other metal combinations,^2^ as we show for PdAu, it falls short to correctly identify how the bond strengths of Au–Au and Pd–Pd change to form Pd–Au bonds on a NP. It should be noted that in this specific bimetallic NP system, the bulk CE values of the two metals are very similar, so effectively capturing the bimetallic bond strength is critical. The new NP method we introduce here to calculate the gamma values overcomes this weakness as it captures bonding information from a gamut of coordination environments compared to the dimer method, where there is only one bond between the two metal atoms.

To evaluate the relative error in calculating the CE values of NPs with the BC model (using the gamma values from the new NP method and the original dimer method) vs DFT, we now include a third metal, Pt, which exhibits a completely different bulk CE value (5.84 eV/atom) than Au (3.81 eV/atom) and Pd (3.89 eV/atom).^42^ Bimetallic NPs consisting of 147 metal atoms and different metal compositions, chemical orderings, and metal combinations of Au, Pt, and Pd were generated using the atomic simulation environment (ASE).^43^ For each combination of metals at each composition there were eight NPs generated with a randomized chemical ordering and two NPs generated with core/shell architecture. Applying the BC model to this data set with the dimer method and the NP method results in root mean squared errors (RMSEs) of 0.331 and 0.110 eV/atom, respectively. The parity between the BC model prediction and the DFT-calculated CE values of the NPs using the dimer method and the NP method is presented in Figure 3a and 3b, respectively. Apart from the significant increase in the accuracy of these calculations as depicted in the RMSE values, it is important to note how the CE of the AuPd bimetallic NPs (blue, green, and purple points in Figure 3) is captured by the new NP method. These specific data show that the BC model is invariant to calculating the CE of the NPs using the dimer method (Figure 3a) and becomes sensitive when using the NP method for the gamma values (Figure 3b). This is the reason why the chemical ordering predictions in Figure 2 completely flip when using the NP method compared to the dimer method. To further improve the model’s performance, a slightly altered definition of the coordination number was incorporated. This definition generalizes the coordination number, introducing noninteger values, to capture interatomic strain effects from the varying atom size within the NP, as shown in eq 7.
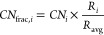
7
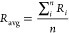
8In eq 7, *R*_avg_ is the averaged radii of the atoms in the NP, *CN*_*i*_ is the original coordination number, and *R*_*i*_ is the radius of atom *i*. The radii were calculated based on DFT-optimized monometallic NPs with 147 atoms as one-half the average bond distance in each geometrically optimized NP. The radii for Au, Pt, and Pd were found to be approximately 1.47, 1.38, and 1.38 Å, respectively. These radii were then used as the cutoffs for bond determination using ASE. Due to the similarity of the radii for Pd and Pt, the *CN*_frac_ will make less of an impact on PdPt NPs. This new coordination number definition resulted in a 11% decrease in the RMSE (eV/atom) overall between the BC model (parametrized using the NP method) and DFT-calculated CE values. Considering that the *CN*_frac_ definition would not influence the 30 PdPt NP energy predictions, due to the similar radii of the two metals, the 11% decrease in the RMSE was a significant improvement. The parity between the BC model (with NP gamma values) incorporating the new CN definition and the DFT results is shown in Figure 3c. Due to the robustness of the *CN*_frac_ implementation in different metal combinations and compositions, the *CN*_frac_ is used in all further results of the BC model. Inherently, *CN*_frac_ captures how the metal composition on the different coordinated sites of the NP will affect the local distances of this specific site. We should note that although the NP CE values fall closer to the DFT-calculated ones with this *CN*_frac_ implementation, the relative ranking of the NPs did not change (Figure 3b and 3c). Thus, the chemical ordering predictions using our GA with either the original CN definition or the *CN*_frac_ did not change much, demonstrating that the relative CE between the NPs is more important to capture stability trends.

## Optimal Chemical Ordering of Multimetallic Nanoparticles

**Figure 4:**
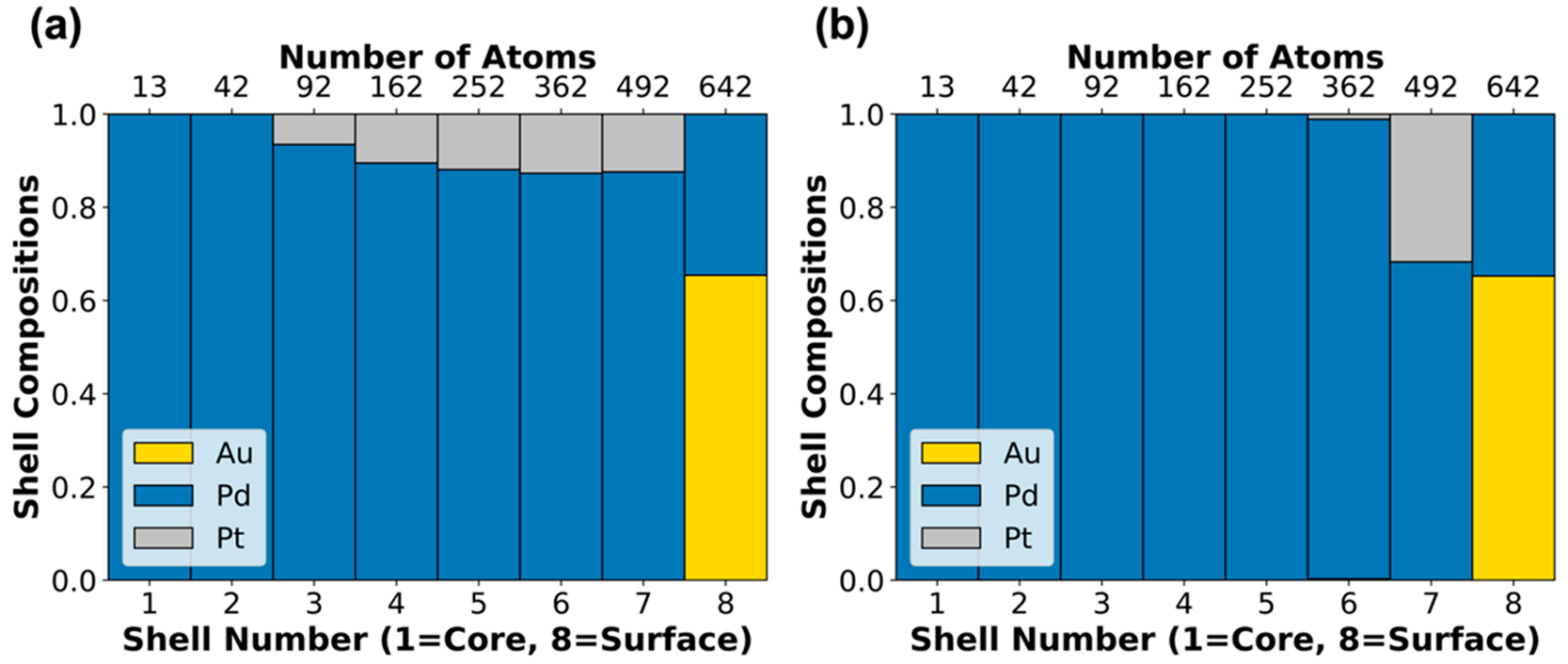
Core to shell radial distribution of Au, Pt, and Pd of an Au_420_Pt_160_Pd_1477_ icosahedral NP. Optimal chemical ordering was found using (a) the GA parametrized with the BC model and (b) MMC simulations (data reproduced from ref (23)).

Multimetallic NPs have versatile catalytic properties that can be attributed to the synergistic effects between the metal alloys.^44^ Therefore, extending the GA to NPs with three or more different metal alloys would be highly valuable. In theory, the BC model can be applied to any multimetallic NP, but to ensure that extending this method past bimetallic NPs is feasible, the accuracy of the GA parametrized by the BC model must be evaluated further. Our previously developed GA code^2^ was updated to allow for chemical ordering predictions using more than two metal types in any given NP, and a mixed integer optimization was run on an AuPdPt NP (test case). The GA was run on the AuPdPt NP containing a total of 2057 atoms until there were at least 10 000 generations without any change in the minimum energy ordering. The choice of 10 000 generations is a hyperparameter in our GA implementation. This hyperparameter should be chosen depending on the size of the feature space (mainly based on the total number of atoms and the number of different elements) and the desired accuracy. The GA results, shown in Figure 4a, demonstrate that in this trimetallic NP, Au still prefers to completely segregate to the surface with Pd covering the remainder of the surface and Pt residing in the subsurface layers of the NP. Mattei et al. used molecular dynamics (MD) and Metropolis Monte Carlo (MMC) to determine the equilibrium composition and ordering of Au, Pt, Pd trimetallic NPs.^23^ The composition of interest that they used was 21% Au, 71% Pd, and 8% Pt. Visualizing the core to shell chemical ordering of the equilibrium NP found by Mattei et al., as seen in Figure 4b, shows that Au will prefer to be on the surface with the rest of the surface being covered by Pd. Pt is shown to be in the first subsurface layer and Pd in every layer distributed from the core to the surface. Comparing the results from our GA simulations (Figure 4a) to Mattei et al.’s MMC results shows that the GA was able to accurately capture important chemical ordering trends such as the chemical composition on the surface of the NP. It is important to note that both method’s predictions were in complete agreement that the first two layers in bulk would entirely consist of Pd, demonstrating that the GA captures not only surface trends but also the thermodynamics in bulk. The only deviation in the core to shell chemical ordering predictions was where the Pt would reside, either solely in the subsurface layer or distributed through the first few subsurface layers of the NP. Further DFT analysis was performed on model NPs, as shown in Figure S1, that alluded to both of these chemical orderings being isoenergetic.

**Figure 5:**
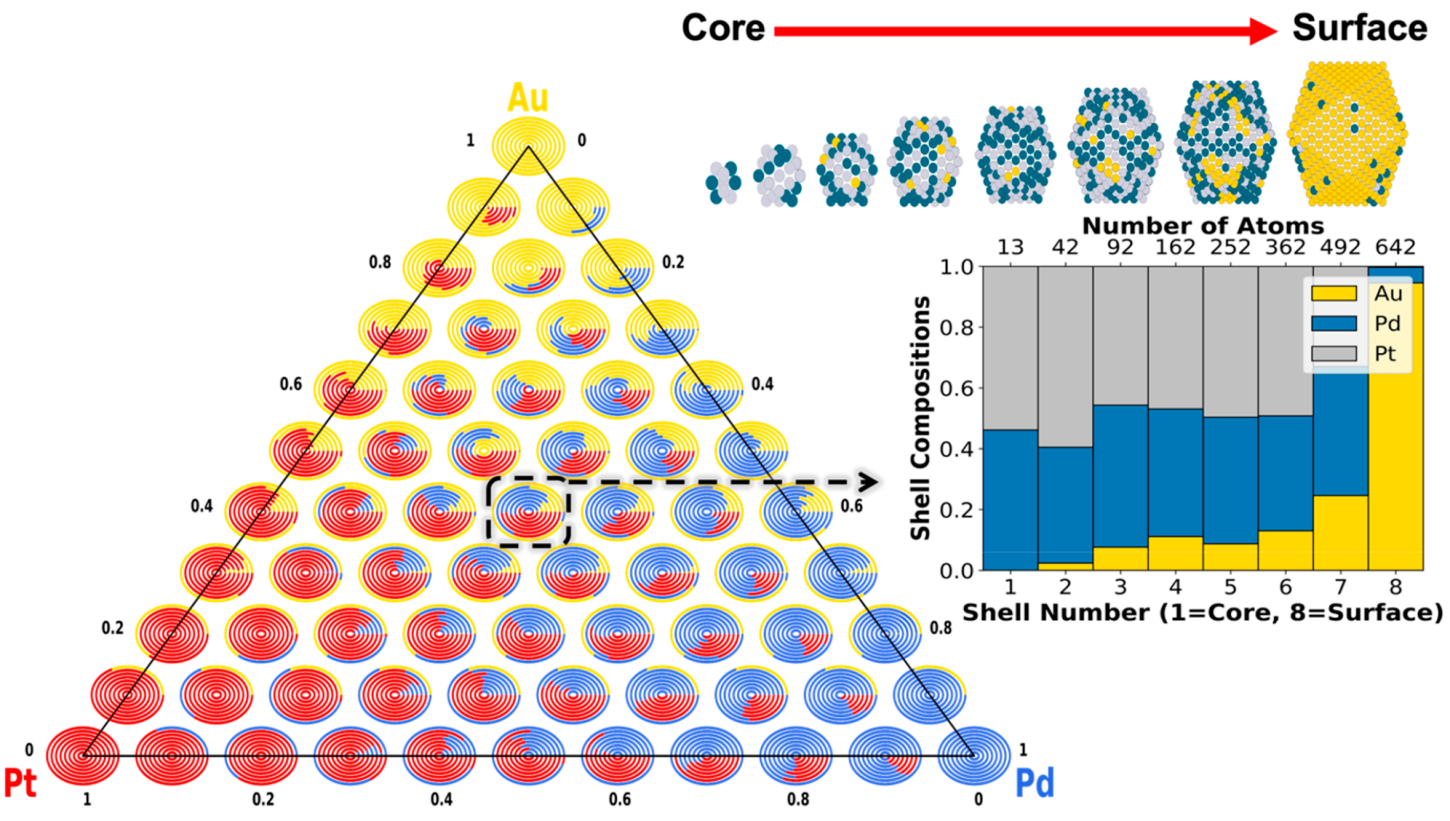
Ternary diagram showing the core (center circle) to shell (outermost circle) composition of 66 different NP compositions. Each circle represents a shell of the NP as depicted in the images to the right of the diagram for the NP with composition 40% Au, 30% Pd, and 30% Pt.

Using the GA we now expand our nanomaterials screening search to explore the NP stability with combinations of the three metals as a function of metal composition, resulting in a comprehensive study of optimal chemical orderings for different Au, Pd, and Pt bi/trimetallic NPs. There were 66 NPs, with 2057 total atoms each (roughly 4.5 nm average diameter), investigated with varying compositions of Au, Pd, and Pt by 10% intervals. The results of this investigation are presented in Figure 5. An important insight is that when the NP is Au or Pd rich, Pt is not likely to be found on the surface of the NP. One example of how this insight can be utilized is the application of AuPt NPs for the reduction of 4-nitrophenol. It has been shown that NP chemical ordering is crucial to this catalytic application due to synergy between Au and Pt atoms when they are both on the surface of the NP.^45^ Using Figure 5 we can deduce that to achieve a thermodynamically highly stable NP with both Au and Pt on the surface of the NP, we should have a composition larger than 50% in Pt (see the left side of ternary phase diagram in Figure 5 consisting of AuPt composition). Additionally, from this ternary diagram it becomes apparent that the total composition of the trimetallic NP is not necessarily depicted on the surface composition of the NP. For example, although one would expect that for the 50% Pd, 40% Au, 10% Pt NP there should be some Pd on the surface, our ternary phase diagram reveals that the surface is fully covered by Au (for this specific NP composition and size). Predictions such as the ones revealed in Figure 5 can be instrumental for both experimental and computational researchers. For instance, they can inform the selection of metals and compositions that may lead to experimentally controlled exposure of specific metals on the NP surface as well as the selection of metal systems with specific chemical ordering, allowing for more realistic simulations (e.g., periodic slabs or metal NPs).

Further information on how to use the GA code for automated chemical ordering predictions can be found at our GitHub repository https://github.com/mpourmpakis/CANELa_NP. The repository contains code to visualize core to shell compositions of multimetallic NPs (similarly to Figures 4 and 5) and generate evenly distributed NPs required to calculate NP gamma values and finally illustrates the use of the previously developed GA from https://github.com/mpourmpakis/ce_expansion using the fractional CN definition shown in eq 7.

## Conclusions and Outlook

Throughout this Account, we have explored optimal chemical ordering trends from bimetallic combinations of Au, Pt, and Pd as well as trimetallic AuPdPt NPs over a wide range of metal compositions. A novel NP method for quantifying synergistic stability effects between two metal alloys was proposed and validated, and a modified CN definition was shown to robustly improve energetic predictions in the developed BC model.^1^ These improvements in the energetic descriptions of NPs were verified with DFT calculations and implemented into a recently developed GA^2^ that automates the discovery of multimetallic NPs with thermodynamically optimal chemical ordering. The BC model^1^ and the GA^2^ have been previously shown to accurately predict excess energy trends that are crucial for understanding nanoalloy mixing. Results from the previous GA chemical ordering predictions on bimetallic NPs of different shapes, sizes, and compositions can be found in http://www.metalnanodb.com. The code developed, which is distributed free of charge on our Github repository, can enable materials design and inform experimental and computational researchers with thermodynamically highly stable multimetallic NPs as a function of metal composition.

On the industrial application side, this GA implementation (with the improvements introduced within this Account) may be used to study the chemical ordering of multimetallic NPs and take advantage of synergistic effects between different metal types to maximize catalytic activity and/or reduce the amount of costly metals in the NP catalyst. The selection of different metals and metal compositions can give rise to NPs with finely tuned chemical composition and ordering on the surface of the NP. Examples of such nanostructures are HEAs due to their wide range of possible active sites^32^ and single-atom alloys (SAAs) which have found tremendous application in the field of catalysis.^46,47^ There have been many models developed in an attempt to screen the vast amount of possible surface configurations to locate optimal HEA catalysts for reactions such as the oxygen reduction (ORR),^48^ CO_2_ reduction (CO_2_RR), and CO reduction (CORR).^49^ These models may benefit from the use of our BC model (parametrized by the NP method) to determine the local thermodynamic stability on the surface of the catalyst (which has been shown to affect the adsorption energy of species such as catalytic intermediates).^3^ With tools like the one we introduce here, one can select metals and compositions of bimetallic NPs that stabilize SAAs. Even further, since catalytically active NPs typically involve precious metals, one can select multimetallic combinations and compositions in a way that the cheapest metal selectively occupies the bulk of the NP and the active (and expensive) metals decorate the NP surface, resulting in designing cheaper, effective catalysts. After using the GA, parametrized by the BC model and gamma values introduced in this Account, to determine the most stable chemical ordering of a NP, the use of the adsorption model developed by Dean et al. could further elucidate the affinity of different sites of a NP to bind catalytic reaction intermediates as a function of metal composition and the local coordination environment.^3^ Moreover, the combination of both could enable the development of optimization algorithms that predict the equilibrium chemical ordering of multimetallic NPs in the presence of a chemical environment and identify chemical species (i.e., ligands, adsorbates, reaction intermediates) that selectively drive the segregation of specific metals on the NP surface. The BC model in its current implementation does not consider environmental effects which can change surface segregation. This is a natural next step in our research to modify the BC model to account for ligands and adsorbates. To this end, there have been recent works that extend cluster expansion methods to capture unique configurational thermodynamics.^50,51^ Then, by knowing the exact structure and metal composition on the NP surface, sophisticated catalytic models can be developed, predicting the catalytic behavior of NP ensembles^4^ that account for, in addition to the NP morphology distribution, the chemical ordering distribution at elevated temperatures. To discern the stability of different particle morphologies and chemical orderings, two-step approaches, such as the one proposed by Yin et al. with AgCu, CuAu, and AuAg nanoclusters, can be implemented with the use of the BC model.^52^ One can first identify the most cohesive bimetallic ordering for a given composition and specific particle shape coupled with a metaheuristic strategy that searches over the space of particle shapes to obtain globally optimal structures (optimizing both chemical ordering and particle morphology). Yin et al. used simulated annealing (SA) to optimize the particle shapes,^52^ and this implementation allows for simulating arbitrary NP shapes by repositioning surface atoms. The scaling relation  (present in the BC model) remains true for NP shapes that display extreme coordination environments. This relationship has been shown to accurately predict the energetics of nonequilibrium NP shapes such as the truncated cuboctahedron.^53^

Finally, the method presented in this Account may be applied to a wide variety of different computational models. In the future, many physics-based machine learning (ML) models will likely be developed as faster and possibly more accurate solutions to a variety of nanocatalysis problems. Some examples of more recent ML applications are the use of neural networks to help predict partial radial distribution functions^54^ and inverse design of NPs to fulfill optical property needs.^55^ Machine learning models may benefit from incorporating the modified CN as an atomic feature. Graph neural network models may benefit by applying the NP method gamma values as interatomic edge features. As ML becomes more prevalent in the field of nanotechnology, simple, physically meaningful descriptors (such as the ones presented in this Account) will be paramount to accelerate and guide the models’ learning curve.

## Supporting Information Available

Physical constants used for the implementation of the BC model, and DFT calculations on trimetallic NPs supporting the chemical ordering predictions (PDF)
