# Supplementary material for: Demystifying the Chemical Ordering of Multimetallic Nanoparticles
Source: Acc Chem Res. 2023 Jan 21;56(3):248–57. doi: 10.1021/acs.accounts.2c00646 (PMC9910050; doi:10.1021/acs.accounts.2c00646)
Supplement: Supplementary file 1 — ar2c00646_si_001.pdf [file ar2c00646_si_001.pdf]

Supporting Information

Demystifying the Chemical Ordering of Multimetallic Nanoparticles

Dennis Johan Loevlie, Brenno Ferreira, and Giannis Mpourmpakis*

Department of Chemical and Petroleum Engineering, University of Pittsburgh, Pittsburgh, Pennsylvania 15261, USA

*Corresponding Author email: gmpourmp@pitt.edu

Table S1: Physical constants, such as bulk cohesive energies of metals (CE), bond dissociation energy (BDE) data of metal dimers, and γ (gamma) values used in the Bond-Centric model.

Symbol	Definition	Value
$CE_{bulk,Au}$	Bulk cohesive energy of Au (eV)	-3.64[1]
$CE_{bulk,Pd}$	Bulk cohesive energy of Pd (eV)	-4.20[2]
$CE_{bulk,Pt}$	Bulk cohesive energy of Pt (eV)	-6.20[2]
BDE_{Au-Au}	Bond dissociation energy of an Au_2 dimer (eV)	2.29[3]
BDE_{Pd-Pd}	Bond dissociation energy of an Pd_2 dimer (eV)	1.03[3]
BDE_{Pt-Pt}	Bond dissociation energy of an Pt_2 dimer (eV)	3.71[3]
BDE_{Au-Pd}	Bond dissociation energy of an Pd_1-Au_1dimer (eV)	1.44[3]
BDE_{Au-Pt}	Bond dissociation energy of an Pt_1-Au_1dimer (eV)	2.425[4]
BDE_{Pd-Pt}	Bond dissociation energy of an Pt_1-Pd_1dimer (eV)	1.948[4]

γ_{Au-Pd}	γ_{Au-Pd} from the dimer method	0.651
γ_{Pd-Au}	γ_{Pd-Au} from the dimer method	1.349
γ_{Au-Pt}	γ_{Au-Pt} from the dimer method	1.810
γ_{Pt-Au}	γ_{Pt-Au} from the dimer method	0.190
γ_{Pd-Pt}	γ_{Pd-Pt} from the dimer method	1.315
γ_{Pt-Pd}	γ_{Pt-Pd} from the dimer method	0.685
γ_{Au-Pd}	γ_{Au-Pd} from the NP method	2.945
γ_{Pd-Au}	γ_{Pd-Au} from the NP method	-0.945
γ_{Au-Pt}	γ_{Au-Pt} from the NP method	0.972
γ_{Pt-Au}	γ_{Pt-Au} from the NP method	1.028
γ_{Pd-Pt}	γ_{Pd-Pt} from the NP method	1.187
γ_{Pt-Pd}	γ_{Pt-Pd} from the NP method	0.813

Comparison between MMC and GA results for AuPdPt trimetallic chemical ordering using DFT.

We evaluated the 2057-atom AuPdPt NP core to shell chemical ordering trends predicted by the MMC and GA, using DFT calculations on smaller, 147-atom model NPs. The 147-atom NPs were used to determine whether a AuPdPt NP is more stable with Pt being solely in the subsurface layer (MMC prediction) or if Pt can be distributed through different subsurface layers of the NP (GA prediction). This was the deviation between the MMC and GA core/shell chemical ordering predictions and the geometrically DFT-optimized electronic energy results in Figure S1 suggest that both chemical orderings are practically isoenergetic (with the GA ordering preferred by 0.38 eV).

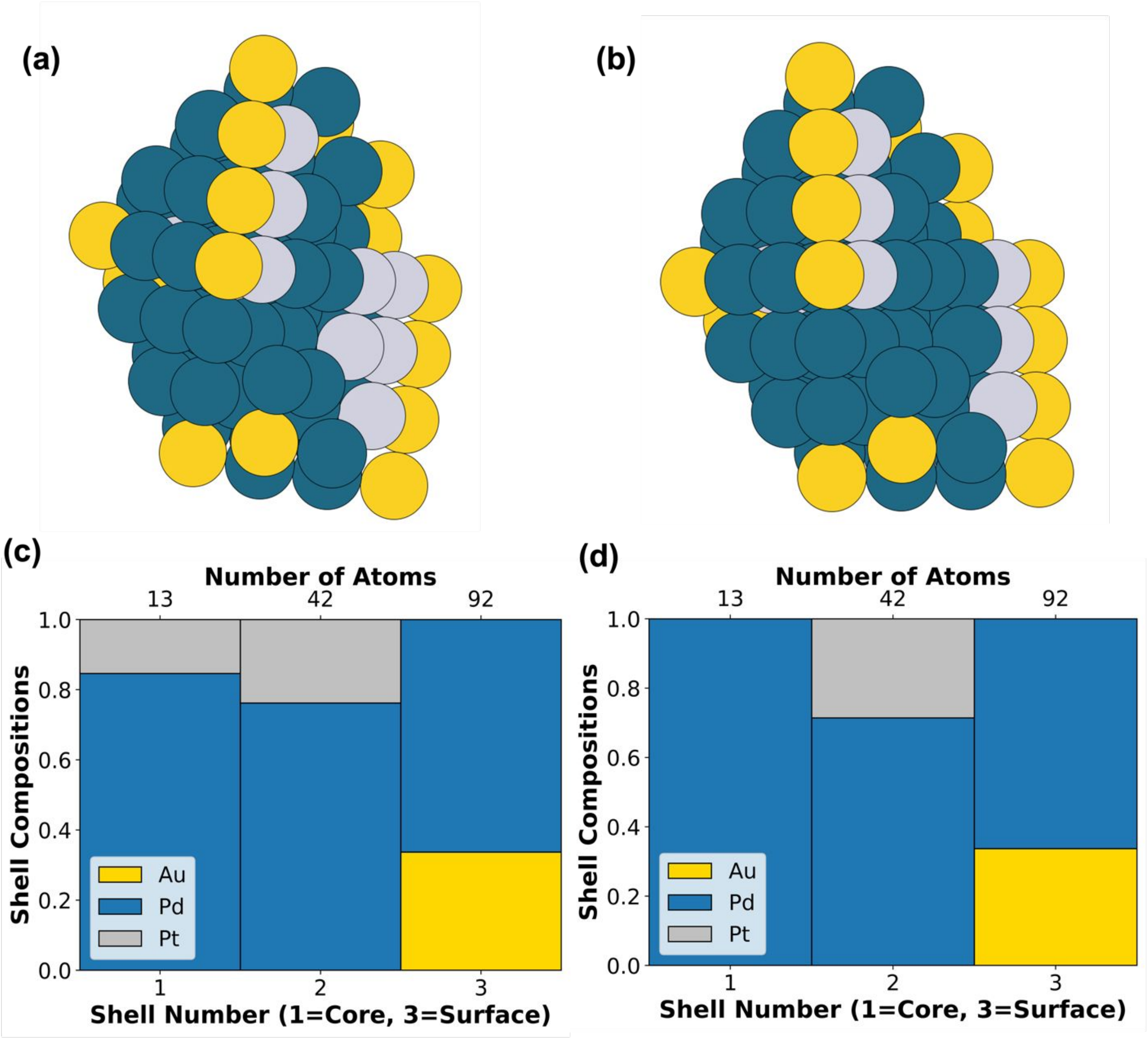

Figure S1: Comparison of DFT calculated NPs (consisting of 147 metal atoms) with similar core/shell chemical ordering distributions as the MMC and GA results in Figure 4. (a), (d) Center cut of NPs with core to shell radial distribution of Au, Pd, and Pt in the NP, corresponding to (c) Pt distributed through the subsurface layers (E=-427,398.58 eV) and (d) Pt localized solely in the first subsurface layer (E=- 427,398.20 eV), respectively. The two structures are isoenergetic.

References

(1) Reckien, W.; Janetzko, F.; Peintinger, M. F.; Bredow, T. Implementation of empirical dispersion corrections to density functional theory for periodic systems. *Journal of computational chemistry* **2012**, *33*, 2023-2031.

(2) Tran, F.; Stelzl, J.; Blaha, P. Rungs 1 to 4 of DFT Jacob's ladder: Extensive test on the lattice constant, bulk modulus, and cohesive energy of solids. *The Journal of Chemical Physics* **2016**, *144*, 204120.

(3) Morse, M. D. Clusters of transition-metal atoms. *Chemical Reviews* **1986**, *86*, 1049-1109.

(4) Miedema, A. R. Model predictions of the dissociation energies of homonuclear and heteronuclear diatomic molecules of two transition metals. *Faraday Symposia of the Chemical Society* **1980**, *14*, 136-148.
